# A novel prognostic model integrating host vulnerability and tumor biology for elderly patients with diffuse large B-cell lymphoma: the BAMAL score

**DOI:** 10.3389/fonc.2026.1720879

**Published:** 2026-02-24

**Authors:** Feiyang Zong, Xudong Zhang, Renjie Hua, Sijun Zhang, Honghan Qiao, Yukai Duan, Qingjiang Chen

**Affiliations:** 1 Department of Oncology, The First Affiliated Hospital, Zhengzhou University, Zhengzhou, China; 2 Henan Academy of Innovations in Medical Science, Zhengzhou, China

**Keywords:** BAMAL score, diffuse large B-cell lymphoma (DLBCL), elderly patients, frailty, host vulnerability, prognostic model

## Abstract

**Background:**

Diffuse large B-cell lymphoma (DLBCL) is the most prevalent subtype of non-Hodgkin lymphoma in adults, with a median age of diagnosis over 65 years. The clinical management of this population presents a growing challenge due to patient heterogeneity, multiple comorbidities, and reduced tolerance to standard immunochemotherapy. Existing prognostic models, such as the International Prognostic Index (IPI), Revised-IPI (R-IPI), and National Comprehensive Cancer Network-IPI (NCCN-IPI), primarily focus on tumor-centric features and often fail to adequately capture patient-specific vulnerability, leading to imprecise risk stratification. This study aimed to develop and internally validate a new, practical prognostic model, the BAMAL score, which integrates established tumor features with novel geriatric assessment parameters and biochemical markers, and to quantitatively compare its predictive performance with traditional models.

**Methods:**

This was a single-center retrospective cohort study of 136 consecutive, newly diagnosed DLBCL patients aged 65 years or older, treated at the First Affiliated Hospital of Zhengzhou University between January 1, 2022, and June 30, 2023. Univariate and multivariate Cox proportional hazards regression analyses were employed to identify independent prognostic factors for overall survival (OS) and progression-free survival (PFS). To account for host vulnerability and competing mortality risks inherent to the elderly, the BAMAL score was constructed using independent prognostic factors identified via backward stepwise multivariate Cox regression (incorporating continuous variables), which were subsequently dichotomized for model formulation. Chi-square tests and trend tests were utilized to evaluate the associations between BAMAL grading, treatment modalities, and therapeutic responses. Discriminatory performance was evaluated using time-dependent Area Under the Curve (AUC) and compared via DeLong’s test, while model fit was assessed using the Akaike Information Criterion (AIC). Additionally, calibration curves were plotted to assess the agreement between predicted and observed survival at 1 and 2 years, and Decision Curve Analysis (DCA) was performed to determine the clinical net benefit of the model for both OS and PFS at these time points.

**Results:**

Multivariate analysis incorporating dichotomized variables identified five independent adverse prognostic factors for OS: bone marrow involvement (HR,2.895), elevated aspartate aminotransferase (AST >40 U/L) (HR,3.132), a modified frailty index (mFI-5) score ≥2 (HR,1.788), age ≥75 years (HR,1.437), and elevated lactate dehydrogenase (LDH >245 U/L) (HR,1.993). The BAMAL score, derived from these five factors, effectively stratified patients into low- (0–1 points), intermediate- (2–3 points), and high-risk (4–5 points) groups, with 2-year OS rates of 84.2%, 58.3%, and 16.7%, respectively. The differences between these groups were highly statistically significant (P<0.001). Notably, a significant association was observed between BAMAL risk stratification and treatment intensity (*P<0.001*). Specifically, the administration of standard immunochemotherapy progressively declined as risk increased, with 82.3% of low-risk patients receiving such regimens compared to only 16.7% of high-risk patients. Therapeutic response analysis revealed a profound gradient, with the High-Risk group exhibiting a complete absence of Complete Responses (0%) and a marked increase in non-evaluable outcomes due to early failure. Although the BAMAL score demonstrated a numerically superior AUC (0.760) compared to NCCN-IPI (0.716) and IPI/R-IPI (0.703) for overall survival, the DeLong test revealed comparable discriminatory power among these models (BAMAL vs. NCCN-IPI: *P=0.358*; vs. IPI/R-IPI: *P=0.268*). Nevertheless, the BAMAL score provided the best model fit as evidenced by the lowest AIC value (366.5). Furthermore, calibration curves demonstrated excellent agreement between predicted and observed probabilities for both OS and PFS at 1 and 2 years. Decision curve analysis (DCA) indicated that while the BAMAL score demonstrated clinical net benefit comparable to NCCN-IPI and IPI/R-IPI for 1-year prediction, it exhibited superior net benefit for 2-year prediction, particularly in the high-risk threshold probability range (>60%), where traditional indices showed limited utility.

**Conclusion:**

The BAMAL score is a novel, practical, and robust prognostic model for elderly DLBCL patients. It successfully integrates features of tumor biology with host-related vulnerability, providing superior predictive accuracy and clinical net benefit compared to standard indices. Crucially, the score offers actionable insights for decision-making: supporting the optimization of standard immunochemotherapy for intermediate-risk patients, while identifying high-risk patients for whom current cytotoxic regimens are futile, underscoring the urgent need for novel, chemotherapy-free therapeutic approaches.

## 1 Introduction

Diffuse large B-cell lymphoma (DLBCL) is the most common subtype of non-Hodgkin lymphoma (NHL), and its incidence rises significantly with age, with a median age at diagnosis typically exceeding 65 years (1). With the global population aging, the management of DLBCL in the elderly has become an increasingly critical clinical challenge (2). This specific patient population frequently presents with multiple comorbidities, diminished physiological reserve, and reduced tolerance to standard immunochemotherapy, which often leads to poorer clinical outcomes compared to younger counterparts (2, 3). Furthermore, older patients are often underrepresented in pivotal clinical trials, resulting in a lack of evidence-based guidance for this highly heterogeneous group (4). Consequently, accurately stratifying risk to balance therapeutic benefits against toxicity is a core dilemma in current clinical practice.

For decades, the International Prognostic Index (IPI) and its subsequent revisions, the Revised-IPI (R-IPI) and the NCCN-IPI, have been the cornerstone tools for risk stratification in DLBCL (5). However, the utility of these models in elderly patients is demonstrably limited for two primary reasons. First, these models were largely developed based on data from a younger patient cohort and over-rely on a subjective measure of physical condition, such as the Eastern Cooperative Oncology Group (ECOG) performance status (PS) (6). While widely used, ECOG PS often underestimates the actual level of frailty in older adults (4). The second limitation of existing models is their insufficient assessment of host-related factors (5). The inherent heterogeneity of the elderly population requires a more comprehensive evaluation of a patient’s biological age and physiological reserve rather than relying solely on chronological age and a basic performance scale (2, 7). It is now widely recognized that integrating a formal Geriatric Assessment (GA) is crucial for managing older cancer patients (4). Tools that quantify frailty and comorbidities, such as the simplified Modified Frailty Index (mFI-5), have proven to be superior to subjective performance status scales (8). The prognostic value of these tools in predicting adverse outcomes has been confirmed across various oncology and surgical disciplines (8–10). Additionally, certain easily accessible biochemical markers that reflect systemic inflammation, nutritional status, and tumor-host interactions have shown new prognostic value (11–13). Elevated aspartate aminotransferase (AST) levels, for instance, have been identified in various malignancies as a negative prognostic factor independent of liver involvement, possibly indicating a more aggressive tumor metabolic phenotype or a broader systemic stress response (14, 15). This study’s central hypothesis is that a new prognostic model, by integrating a validated frailty assessment tool (mFI-5) and a novel biochemical marker (AST) with established tumor-related factors, can create a more comprehensive “tumor-host” assessment system. This new system is expected to provide superior prognostic stratification compared to current models. Therefore, this study aimed to identify independent prognostic factors for OS in a real-world cohort of newly diagnosed elderly DLBCL patients and to develop and internally validate a novel prognostic scoring system, the BAMAL score, to provide a more accurate and practical decision-making tool for their clinical management.

## 2 Patients and methods

### 2.1 Study design and patient population

This was a single-center, retrospective cohort study conducted at the First Affiliated Hospital of Zhengzhou University. We continuously enrolled 136 patients who were newly diagnosed with DLBCL and were aged 65 years or older between January 1, 2022, and June 30, 2023. The primary study endpoint was overall survival (OS), defined as the time from the initiation of treatment to death from any cause. Progression-free survival (PFS) was evaluated as a secondary endpoint, defined as the time from the initiation of treatment to the first occurrence of either disease progression or death from any cause. The inclusion criteria were as follows: (1) age≥65 years at diagnosis; (2) pathological confirmation of DLBCL according to the 2022 World Health Organization (WHO) classification of tumors of hematopoietic and lymphoid tissues; and (3) availability of essential baseline clinicopathological and follow-up data, with mandatory documentation of serum lactate dehydrogenase (LDH) levels for risk stratification. Patients were excluded if they had (1) primary central nervous system lymphoma; (2) DLBCL that transformed from a pre-existing indolent lymphoma; (3) concurrent human immunodeficiency virus (HIV) infection or other severe immunodeficiency diseases; or (4) received best supportive care only without any systemic anti-lymphoma treatment; (5) High-Grade B-Cell Lymphoma (HGBL) defined according to the 2022 WHO Classification of Hematolymphoid Tumors (i.e., DLBCL with concurrent MYC and BCL2 rearrangements) (16); (6) Active liver disease, including active viral hepatitis or decompensated cirrhosis, to ensure that elevated AST levels reflect tumor metabolic activity or systemic stress rather than primary hepatic pathology. The study protocol was approved by the Institutional Review Board and Medical Ethics Committee of the First Affiliated Hospital of Zhengzhou University (No. 2022-KY-0869-001) and was conducted in accordance with the Declaration of Helsinki.

### 2.2 Data collection and variable definitions

All clinical data were retrospectively collected from the electronic medical record system. Baseline information gathered included demographic characteristics, clinical and pathological variables, laboratory test results, and geriatric assessment-related variables. A key variable was the Modified Frailty Index (mFI-5), which was calculated based on five indicators: non-independent functional status, diabetes, chronic obstructive pulmonary disease or pneumonia, congestive heart failure, and hypertension requiring medication (17). Each indicator present was assigned one point, with the total score ranging from 0 to 5 points. Consistent with prior literature, patients with an mFI-5 score of≥2 were defined as frail (8). All continuous variables were dichotomized using clinically recognized cutoff values or thresholds established in the literature, including age (<75 years vs. ≥75 years), LDH (≤245 U/L vs. >245 U/L), AST (≤40 U/L vs. >40 U/L), and ALT (≤40 U/L vs. >40 U/L (18–20). Additionally, baseline IPI, R-IPI, and NCCN-IPI scores were calculated for each patient based on their established definitions (5, 21). Other study variables were defined as follows. Clinical staging was determined using the Ann Arbor system, specifically based on the 2014 Lugano classification (the Lugano modification of the Ann Arbor staging system) to evaluate the extent of Hodgkin and non-Hodgkin lymphomas (22). Tumor biology was characterized via immunohistochemistry (IHC): Double-expressor (DE) status was identified as MYC expression ≥40% and BCL-2 expression ≥50% in tumor cells (23); The Ki-67 proliferation index was quantified as the percentage of positive tumor cells, with positivity defined as an index ≥70%, a threshold established in previous studies to identify patients with highly aggressive disease and poor clinical outcomes (24); and Epstein-Barr virus (EBV) status was assessed by EBER-ISH, with positivity defined as positive nuclear staining in tumor cells (16, 25). Bulky disease was defined as a maximum tumor diameter ≥7.5 cm, consistent with the criteria established in previous large-scale clinical trials (26).The maximum standardized uptake value (SUVmax) was recorded as the highest activity within a single voxel in the selected volume of interest, reflecting the most metabolically active region of the lesion (27). Laboratory and nutritional parameters were categorized using established cut-off values. Hematological thresholds were defined based on standard clinical practice and established guidelines for the Chinese population: anemia (Hemoglobin < 120g/L for males; < 110g/L for non-pregnant females), thrombocytopenia (platelets < 100 × 10^9^/L), which serves as a critical cutoff for chemotherapy administration and dose intensity in lymphoma management, and leukopenia (white blood cell count < 4.0 × 10^9^/L) (28–30). Additionally, elevated CRP (> 5 mg/L), elevated creatinine (> 115μmol/L), hypoalbuminemia (albumin < 35 g/L), and hypoglobulinemia (globulin < 20 g/L) were defined (31–34). Comorbidities were quantified using the Charlson Comorbidity Index (CCI), which weights 19 underlying conditions to predict mortality risk (35); subsequently, the age-adjusted CCI (aCCI) was calculated by incorporating an age-based correction factor into the standard CCI score to better stratify risk in this elderly cohort (36). Polypharmacy was defined as the concurrent use of five or more medications (37). Systemic inflammatory and nutritional indices included the lymphocyte-to-monocyte ratio (LMR≤ 3.0) (38), platelet-to-lymphocyte ratio (PLR ≥ 150) (39), and the neutrophil-to-lymphocyte ratio (NLR), the latter being categorized as ≤1 (physiological), 1-3 (normal), 3-5 (mild inflammation), and > 5 (severe inflammation) (40, 41). Nutritional status was assessed via the Prognostic Nutritional Index (PNI) and the Geriatric Nutritional Risk Index (GNRI). The Prognostic Nutritional Index (PNI) was calculated using the formula: 10 × serum albumin (g/dL) + 5 × absolute lymphocyte count (10^9/L). Patients were stratified into four nutritional risk categories: normal (> 50), mild (45–50), moderate (40–44), and severe (< 40), with lower scores indicating higher risk of postoperative complications and poor prognosis (42). Similarly, the Geriatric Nutritional Risk Index (GNRI), a specialized assessment tool for the elderly, was calculated using the formula: 1.489 × serum albumin (g/L) + 41.7 × (actual weight/ideal weight), Lower GNRI values indicate higher nutritional risk and a worse prognosis, with risk levels categorized as none (> 98), mild (92-98), moderate (82-91), and severe (< 82) (43). Notably, the optimal cut-off values for LMR, PLR, NLR, PNI, and GNRI were determined based on existing literature rather than receiver operating characteristic (ROC) curve analysis.

### 2.3 Treatment regimens and follow-up

The specific first-line treatment regimen received by each patient was recorded and categorized into two groups: (1) standard immunochemotherapy (including standard-dose R-CHOP[Rituximab plus Cyclophosphamide, Doxorubicin, Vincristine, and Prednisone] and R-mini-CHOP [recommended by National Comprehensive Cancer Network [NCCN] guidelines for elderly/frail patients]) (44); and (2) alternative regimens (including systemic palliative strategies such as R-monotherapy, R-CVP,BR, or R² [rituximab plus lenalidomide] for patients deemed unfit for anthracyclines). Patients receiving best supportive care alone were excluded from the analysis to minimize selection bias. Treatment response was evaluated according to the Lugano 2014 classification criteria (22). Outcomes were categorized as Complete Response (CR), Partial Response (PR), Stable Disease (SD), Progressive Disease (PD), or Not Evaluable (NE). Patients were followed up via telephone calls or outpatient clinic visits, with the final follow-up date being November 30, 2024, or the date of the patient’s death.

### 2.4 Statistical analysis

All statistical analyses were conducted using SPSS software, version 26.0 (IBM Corp., Armonk, NY, USA) and R software, version 4.3.1 (The R Foundation for Statistical Computing, Vienna, Austria). Categorical variables were compared using the Chi-square test or Fisher’s exact test, while continuous variables were analyzed using the Mann-Whitney U test or t-test, as appropriate. Kaplan-Meier curves were plotted to illustrate survival, and between-group comparisons were performed using the Log-Rank or Breslow (Generalized Wilcoxon) test, depending on the temporal characteristics of the survival differences. Univariate Cox proportional hazards regression was initially performed to screen for variables with a significant association. The final set of independent prognostic factors for OS and PFS was then selected using a backward stepwise multivariate Cox proportional hazards model, incorporating continuous variables to maximize statistical power. For the construction of the scoring system, these factors were subsequently dichotomized to derive integer-based risk scores. The predictive performance of the models was evaluated and compared using time-dependent Area Under the Curve (AUC) and Akaike Information Criterion (AIC), and the statistical significance of the differences in AUCs between models was assessed using the DeLong test. Furthermore, calibration curves were generated to assess the agreement between predicted and observed survival at 1 and 2 years, and Decision Curve Analysis (DCA) was performed to quantify the clinical net benefit. All analyses were two-sided, and a P value of <0.05 was considered to indicate statistical significance.

## 3 Results

### 3.1 Patient baseline characteristics and survival outcomes

**Figure 1:**
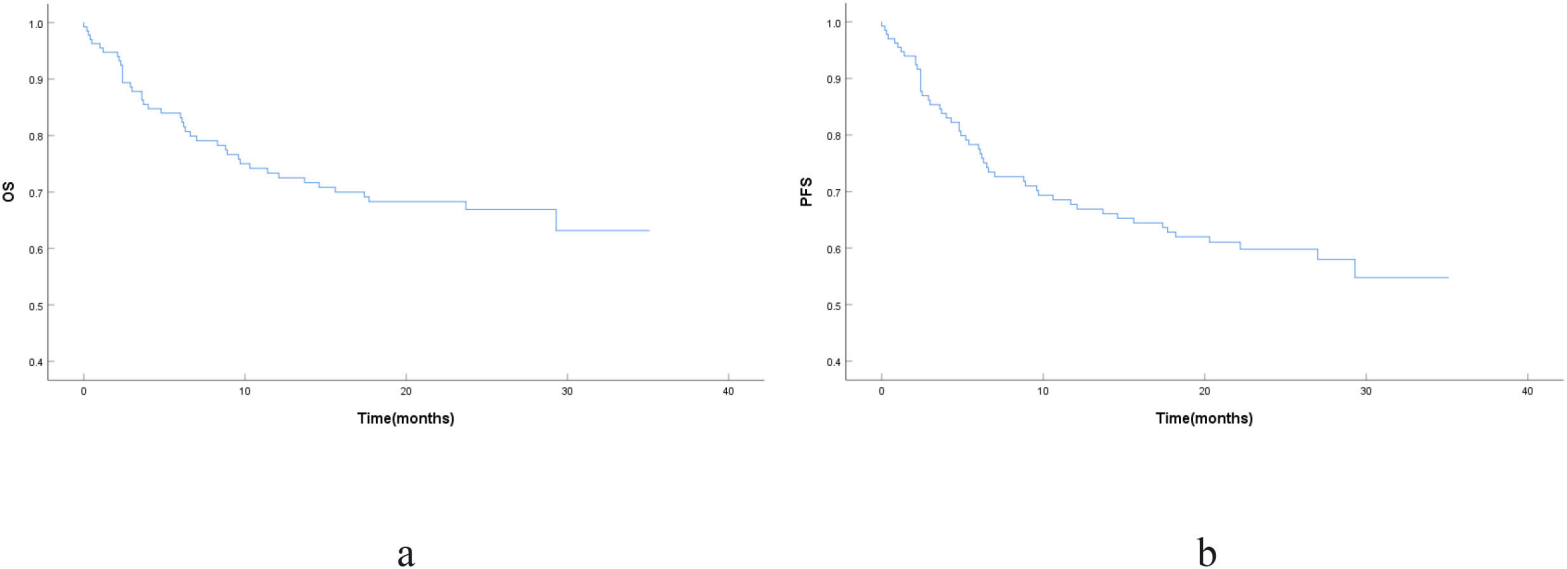
Kaplan-meier curves for the entire patient cohort. **(a)** Overall Survival (OS); **(b)** Progression-Free Survival (PFS).

A total of 136 patients were included in the study, with a median age of 72 years (range: 66–92 years) and 49.3% being male. Regarding geriatric-related features, 27.4% of patients had an ECOG PS score of≥2, and 39.0% were classified as frail according to the mFI-5 score (≥2). The median follow-up time was 25.1 months (95%CI, 22.4–27.8), and the median OS and PFS were not reached. For the OS analysis, 94 patients (69.1%) were censored (alive at last follow-up), and 42 deaths (30.9%) were observed. Regarding PFS, 84 patients (61.8%) were censored (alive and progression-free), while 52 events (38.2%) were recorded. The estimated 2-year OS and PFS rates for the cohort were 66.9% and 59.8%, respectively (**Figure 1**). Notably, while lymphoma progression was the leading cause of death (64.3%), a substantial proportion, 35.7%, was attributed to non-lymphoma-related causes, with pulmonary infection being the most common (26.2%), followed by heart failure and multiple organ dysfunction. This finding underscores the significant “competing risk of mortality”in elderly patients, where physiological vulnerability, in addition to the cancer itself, accounts for a considerable number of deaths.

### 3.2 Prognostic factor analysis and BAMAL model construction

**Figure 2:**
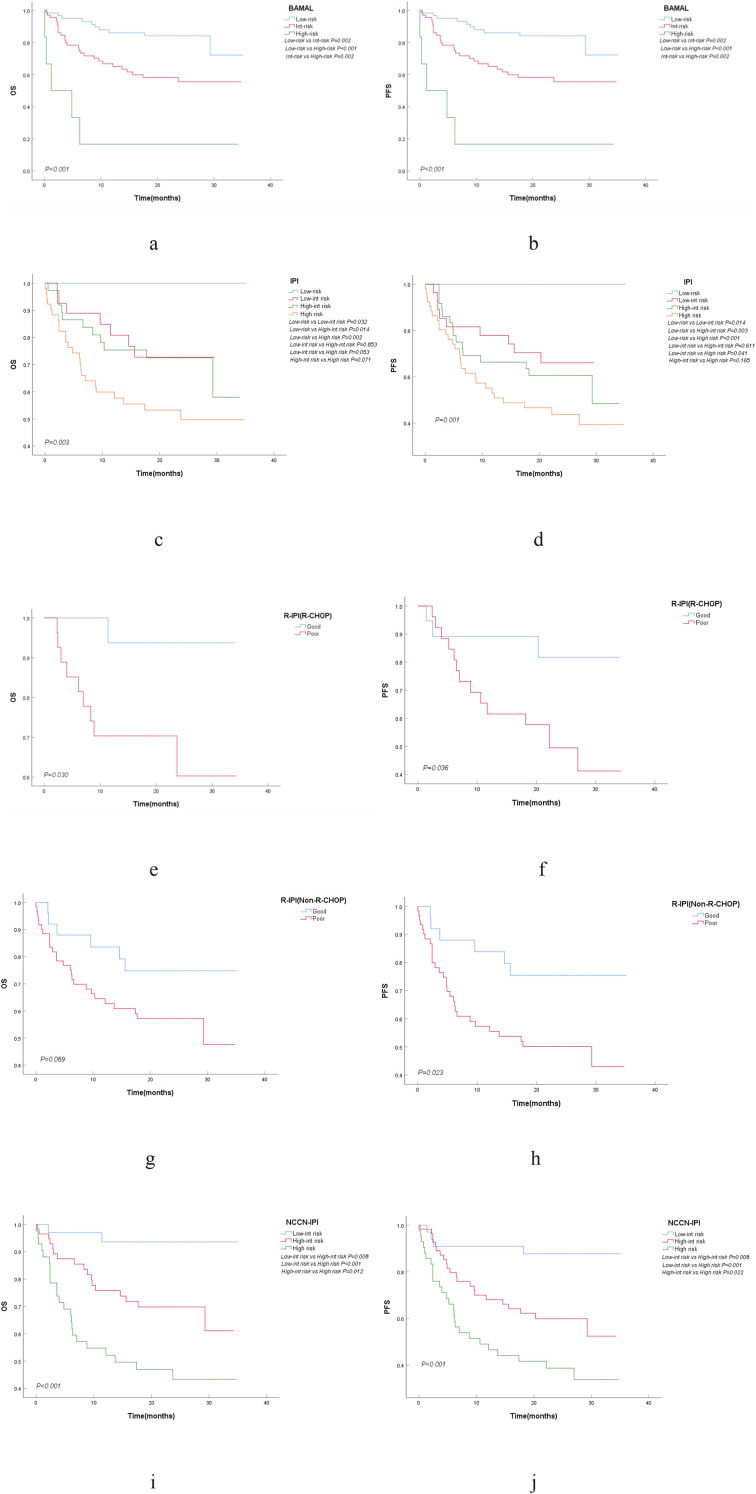
Survival probabilities of the elderly DLBCL cohort. Kaplan-Meier curves show overall survival (OS) and progression-free survival (PFS) stratified by BAMAL score **(a, b)**, International Prognostic Index (IPI) score **(c, d)**, Revised-IPI (R-IPI) score in patients receiving R-CHOP **(e, f)** and non-R-CHOP regimens **(g, h)**, and National Comprehensive Cancer Network-IPI (NCCN-IPI) score **(i, j)**. OS, Overall Survival; PFS, Progression-Free Survival; R-CHOP, Rituximab plus Cyclophosphamide, Doxorubicin, Vincristine, and Prednisone.

In the univariate analysis, hemoglobin level, platelet count, bone marrow involvement, Ann Arbor stage, ECOG PS, ALT, AST, albumin, mFI-5, aCCI, age, LMR, and LDH levels were all significantly associated with poorer OS (*P<0.05*). To identify the most robust independent predictors of survival while minimizing information loss, a multivariate Cox regression analysis was performed using a backward stepwise selection method. In this model, Age, LDH, and mFI-5 were analyzed as continuous variables, while AST and Bone Marrow Involvement were analyzed as categorical variables. As shown in **Table 1**, all five factors maintained high statistical significance (*P < 0.05*) in their continuous/original forms, confirming their independent prognostic value driven by biological gradients. To facilitate the construction of the clinically applicable BAMAL scoring system, these five identified independent predictors were dichotomized based on optimal cut-off values (Age ≥75 years, LDH >245 U/L, mFI-5 ≥2) and re-evaluated in a categorical Cox model. In this categorical analysis, Bone Marrow Involvement (HR 2.895, 95% CI: 1.540–5.444; *P=0.001*) and Elevated AST (HR 3.132, 95% CI: 1.422–6.901; *P=0.005*) maintained robust significance. Notably, the dichotomized LDH (HR 1.993, 95% CI: 0.958–4.149; *P=0.065*) and mFI-5 (HR 1.788, 95% CI: 0.958–3.338; *P=0.068*) showed borderline significance, and Age ≥75 (HR 1.437, 95% CI: 0.754–2.739; *P=0.270*) lost statistical significance. This attenuation in *P*-values is attributable to the statistical power reduction inherent in dichotomizing continuous variables within a limited cohort size. However, given their solid validation in the continuous model (**Table 1**) and the consistent directionality of risk (all HR > 1.400), a uniform scoring strategy was adopted to prioritize clinical simplicity. Instead of weighting based on regression coefficients, each of the five risk factors was assigned a score of 1 point. This approach ensures the model’s ease of use at the bedside without sacrificing biological comprehensiveness. Overall Survival (OS) was selected as the primary endpoint for model construction to account for the substantial burden of non-lymphoma mortality and host frailty in this elderly population. The final BAMAL score is an acronym derived from these five risk factors: Bone marrow involvement, AST elevation, mFI-5 ≥2, Age ≥75, and LDH elevation (Total score: 0–5) (**Table 2**). The score stratified patients into low- (0–1 points), intermediate- (2–3 points), and high-risk (4–5 points) groups, with highly significant differences in 2-year OS rates of 84.2%, 58.3%, and 16.7%, respectively (*P<0.001*) (**Table 3**, **Figure 2**). Importantly, while ECOG PS was a significant prognostic factor in the univariate analysis, it lost its independent prognostic value in the multivariate model. This suggests that the physiological status information reflected by ECOG PS may have been more objectively and comprehensively captured by the mFI-5 score. For PFS, multivariate analysis identified four independent prognostic factors: double-expressor status (HR 2.233, *P=0.009*), Ann Arbor stage (HR 2.989, *P=0.008*), elevated ALT (HR 5.226, *P=0.001*), and elevated LDH (HR 1.001, *P=0.030*) (**Table 1**). This finding indicates that the factors influencing disease progression are not entirely identical to those determining overall survival, with the latter being more heavily influenced by host-related factors.

**Table 1:** Univariate and backward stepwise multivariate Cox regression analysis identifying independent prognostic factors for overall survival.

Variable category	N(%)/Median(range)	Overall survival (OS)		Progression-free survival (PFS)	
		Univariate HR (95% CI; p value)	Multivariate HR (backward stepwise;95% CI; p value)	Univariate HR (95% CI; p value)	Multivariate HR (backward stepwise;95% CI; p value)
Patient demographics and clinical features					
Age	72(66-92)	**1.059(1.003-1.118; *P=0.039*)**	**1.075(1.013-1.141; *P=0.017*)**	1.040(0.989-1.094; *P=0.127*)	
Male	67/136(49.3%)	0.949(0.518-1.741; *P=0.867*)		1.070(0.621-1.844; *P=0.807*)	
Unmarried	14/136(10.3%)	1.097(0.688-1.751; *P=0.697*)		1.115(0.728-1.707; *P=0.617*)	
Smoking History	19/136(14.0%)	0.546(0.195-1.531; *P=0.250*)		1.043(0.491-2.215; *P=0.913*)	
B Symptoms	46/136(33.8%)	1.321(0.709-2.463; *P=0.381*)		1.199(0.682-2.109; *P=0.529*)	
ECOG PS≥2	37/135(27.4%)	**2.569(1.389-4.753; *P=0.003*)**		**2.007(1.139-3.535; *P=0.016*)**	
Manual Laborer	64/101(63.4%)	0.765(0.362-1.616; *P=0.482*)		0.936(0.484-1.810; *P=0.844*)	
Hospital Stay > 14 days	15(3-49)	1.008(0.978-1.040; *P=0.592*)		1.004(0.977-1.033; *P=0.759*)	
Disease and tumor biology					
Ann Arbor Stage III/IV	84/136(61.8%)	**2.439(1.166-5.102; *P=0.018*)**		**2.265(1.187-4.322; *P=0.013*)**	**2.989(1.336-6.685;*P=0.008*)**
Double Expressor Status	45/118(38.1%)	1.801(0.917-3.537; *P=0.088*)		**1.875(1.036-3.393; *P=0.038*)**	**2.233(1.218-4.095;*P=0.009*)**
Bone Marrow Involvement	38/135(28.1%)	**2.765(1.506-5.075; *P=0.001*)**	**2.955(1.495-5.841; *P=0.002*)**	**2.100(1.205-3.662; *P=0.009*)**	
CNS Involvement	3/135(2.2%)	1.718(0.414-7.120; *P=0.456*)		**3.341(1.032-10.811; *P=0.044*)**	
≥4 Lymph Node Sites	101/134(75.4%)	1.749(0.776-3.939; *P=0.177*)		2.044(0.962-4.344; *P=0.063*)	
≥2 Extranodal Sites	84/136(61.8%)	1.303(0.684-2.483; *P=0.421*)		1.732(0.948-3.166; *P=0.074*)	
Bulky Disease (>7.5 cm)	13/130(10.0%)	0.494(0.119-2.053; *P=0.332*)		0.372(0.090-1.534; *P=0.171*)	
Double Hit (c-MYC+, BCL6+)	3/83(3.6%)	2.182(0.293-16.261; *P=0.446*)		2.173(0.294-16.061; *P=0.447*)	
ABC(Hans Classification)	94/135(69.6%)	1.331(0.667-2.657; *P=0.417*)		1.261(0.682-2.333; *P=0.460*)	
EBRB	10/131(7.6%)	0.981(0.302-3.182; *P=0.974*)		0.726(0.226-2.333; *P=0.590*)	
Ki-67	80(40-95)	1.013(0.984-1.044; *P=0.377*)		1.013(0.987-1.041; *P=0.321*)	
P53	70(3-100)	1.012(0.993-1.030; *P=0.220*)		1.001(0.986-1.017; *P=0.847*)	
SUVmax	23.1(6-72)	1.009(0.984-1.034; *P=0.485*)		1.011(0.991-1.032; *P=0.290*)	
Comorbidities and geriatric assessment					
Hypertension	58/136(42.6%)	1.552(0.847-2.844; *P=0.155*)		1.177(0.680-2.038; *P=0.560*)	
Diabetes	23/136(16.9%)	1.739(0.855-3.540; *P=0.127*)		1.139(0.555-2.338; *P=0.722*)	
Coronary Artery Disease	20/136(14.7%)	1.605(0.768-3.356; *P=0.208*)		1.051(0.495-2.233; *P=0.897*)	
mFI-5	1(0-3)	**1.666(1.192-2.329; *P=0.003*)**	**1.813(1.293-2.542; *P=0.001*)**	1.318(0.960-1.809; *P=0.088*)	
aCCI	5(4-7)	**1.481(1.182-1.857; *P=0.001*)**		**1.302(1.033-1.642; *P=0.025*)**	
Polypharmacy	7/135(5.2%)	2.209(0.788-6.197; *P=0.132*)		1.242(0.387-3.986; *P=0.716*)	
Laboratory and inflammatory markers					
Anemia	55/136(40.4%)	**2.255(1.227-4.145; *P=0.009*)**		**1.917(1.112-3.307; *P=0.019*)**	
Thrombocytopenia	12/136(8.8%)	**2.498(1.107-5.633; *P=0.027*)**		1.886(0.849-4.187; *P=0.119*)	
Leukopenia	19/136(14.0%)	0.972(0.406-2.326; *P=0.949*)		0.948(0.444-2.023; *P=0.890*)	
Elevated LDH	233(99-1993)	**1.001(1.001-1.001; *P<0.001*)**	**1.001(1.000-1.001; *P=0.001*)**	**1.001(1.000-1.001; *P<0.001*)**	**1.001(1.000-1.001;*P=0.030*)**
Elevated CRP	58/83(69.9%)	1.377(0.581-3.260; *P=0.467*)		1.582(0.678-3.691; *P=0.288*)	
Elevated ALT	8/136(5.9%)	**3.562(1.391-9.122; *P=0.008*)**		**4.142(1.747-9.821; *P=0.001*)**	**5.226(2.000-13.654;*P=0.001*)**
Elevated AST	15/136(11.0%)	**4.093(1.947-8.606; *P<0.001*)**	**2.801(1.140-6.880; *P=0.025*)**	**3.105(1.506-6.403; *P=0.002*)**	
Hypoglobulinemia	11/136(8.1%)	1.073(0.328-3.509; *P=0.907*)		2.293(0.555-9.472; *P=0.252*)	
Hypoalbuminemia	42/136(30.9%)	**2.102(1.133-3.898; *P=0.018*)**		1.700(0.966-2.993; *P=0.066*)	
Elevated Creatinine	4/136(2.9%)	0.048(0.000-367.680; *P=0.506*)		0.797(0.110-5.776; *P=0.822*)	
LMR	2.9(0.3-8.8)	**0.746(0.601-0.927; *P=0.008*)**		0.885(0.748-1.047; *P=0.155*)	
PLR	140.1(7.0-454.1)	1.000(0.998-1.001; *P=0.779*)		1.000(0.998-1.001; *P=0.744*)	
NLR	2.7(0.7-10.4)	1.024(0.924-1.134; *P=0.653*)		1.004(0.913-1.105; *P=0.929*)	
PNI	44.4(26.0-139.0)	1.004(0.973-1.035; *P=0.819*)		0.976(0.944-1.009; *P=0.157*)	
GNRI	96.9(36.0-116.2)	0.980(0.959-1.002; *P=0.079*)		0.984(0.964-1.004; *P=0.120*)	

Continuous variables (Age, LDH, mFI-5) were entered into the stepwise model in their continuous forms to maximize statistical power and retain information. The final selected variables are shown with their respective Hazard Ratios. HR, Hazard Ratio; CI, Confidence Interval; ECOG PS, Eastern Cooperative Oncology Group Performance Status; CNS, Central Nervous System; EBER, Epstein-Barr virus-Encoded small RNA; SUVmax, Maximum Standardized Uptake Value; mFI-5, 5-item Modified Frailty Index; aCCI, Age-adjusted Charlson Comorbidity Index; LDH, Lactate Dehydrogenase; CRP, C-Reactive Protein; ALT, Alanine Aminotransferase; AST, Aspartate Aminotransferase; LMR, Lymphocyte-to-Monocyte Ratio; PLR, Platelet-to-Lymphocyte Ratio; NLR, Neutrophil-to-Lymphocyte Ratio; PNI, Prognostic Nutritional Index; GNRI, Geriatric Nutritional Risk Index. Statistically significant values (*P < 0.05*) are formatted in bold.

**Table 2:** Comparison of factors and scoring criteria in different prognostic models for elderly DLBCL.

Adverse prognostic factor	BAMAL score	IPI	R-IPI	NCCN-IPI
Core clinical metrics				
Age	≥ 75 years	> 60 years	> 60 years	>40 - ≤60 years (1 point) | >60 - ≤75 years (2 points) | > 75 years (3 points)
ECOG performance status	–	≥ 2	≥ 2	≥ 2
LDH level	≥ 245 U/L	> Upper limit of normal	> Upper limit of normal	>1 - ≤3 × ULN (1 point) | >3 × ULN (2 points)
Ann arbor stage	–	III/IV	III/IV	III/IV
Extranodal involvement				
Extranodal organ involvement	–	> 1 site	> 1 site	Involvement of Specific High-Risk Organs
Bone marrow involvement	Yes	Counted as an extranodal site	Counted as an extranodal site	Yes (As a specific high-risk organ)
Geriatric/host-related metrics				
Frailty status	mFI-5 ≥ 2	–	–	–
AST level	> 40 U/L	–	–	–

IPI, International Prognostic Index; R-IPI, Revised-IPI; NCCN-IPI, National Comprehensive Cancer Network-IPI; LDH, Lactate Dehydrogenase; ULN, Upper Limit of Normal; mFI-5, 5-item Modified Frailty Index; AST, Aspartate Aminotransferase.

**Table 3:** Risk stratification and survival outcomes of different prognostic models in elderly DLBCL patients.

Prognostic Assessment Model	N(%)		2-Year OS(%)		2-Year PFS(%)	
BAMAL						
Low Risk (0-1)	62/136 (45.6%)		84.20%		71.20%	
Intermediate Risk (2-3)	68/136 (50.0%)		58.30%		52.90%	
High Risk (4-5)	6/136 (4.4%)		16.70%		16.70%	
IPI						
Low Risk (0-1)	16/136 (11.8%)		–		–	
Low-Intermediate Risk (2)	30/136 (22.1%)		72.70%		66.10%	
High-Intermediate Risk (3)	38/136 (27.9%)		72.50%		60.60%	
High Risk (4-5)	52/136 (38.2%)		49.70%		43.70%	
NCCN-IPI						
Low Risk (0-1)	0/136 (0.0%)		–		–	
Low-Intermediate Risk (2-3)	35/136 (25.7%)		93.60%		87.70%	
High-Intermediate Risk (4-5)	59/136 (43.4%)		69.80%		59.90%	
High Risk (≥ 6)	42/136 (30.9%)		43.30%		38.70%	
R-IPI						
	R-CHOP	Non-R-CHOP	R-CHOP	Non-R-CHOP	R-CHOP	Non-R-CHOP
Very Good (0)	0/49 (0.0%)	0 (0.0%)	–	–	–	–
Good (1-2)	20/49 (40.8%)	26/87 (29.9%)	93.80%	74.80%	81.70%	75.40%
Poor (3-5)	29/49 (59.2%)	61/87 (70.1%)	60.30%	57.20%	49.50%	50.10%

OS, Overall Survival; PFS, Progression-Free Survival; IPI, International Prognostic Index; R-IPI, Revised International Prognostic Index; NCCN-IPI, National Comprehensive Cancer Network International Prognostic Index; R-CHOP, Rituximab plus Cyclophosphamide, Doxorubicin, Vincristine, and Prednisone.

### 3.3 Treatment regimens, survival analysis, and response rates

Treatment regimens were categorized based on curative intent into standard immunochemotherapy (including standard-dose R-CHOP and R-mini-CHOP) and Alternative Regimens (including anthracycline-sparing protocols such as R-CVP, BR, R², or palliative monotherapy). A highly significant linear association was observed between BAMAL risk stratification and treatment intensity (*P < 0.001*; **Table 4**). The utilization of standard immunochemotherapy decreased progressively as risk increased, with 82.3% (51/62) of Low-Risk patients receiving such regimens, compared to 63.2% (43/68) in the Intermediate-Risk group and only 16.7% (1/6) in the High-Risk group. This distribution reflects a real-world tendency toward treatment de-escalation for patients perceived as having high host vulnerability.

**Table 4:** Association between BAMAL risk stratification and first-line treatment modalities.

BAMAL risk group	Standard immunochemotherapy(n=95)	Alternative regimens (n=41)	*P* for trend
Low Risk(n=62)	51(82.3%)	11(17.7%)	***P<0.001***
Intermediate Risk(n=68)	43(63.2%)	25(36.8%)	
High Risk(n=6)	1(16.7%)	5(83.3%)	

*P* values were calculated using the Chi-square test or Fisher’s exact test to assess the trend across risk groups.

Bold values indicate statistical significance (P < 0.05).

**Figure 3:**
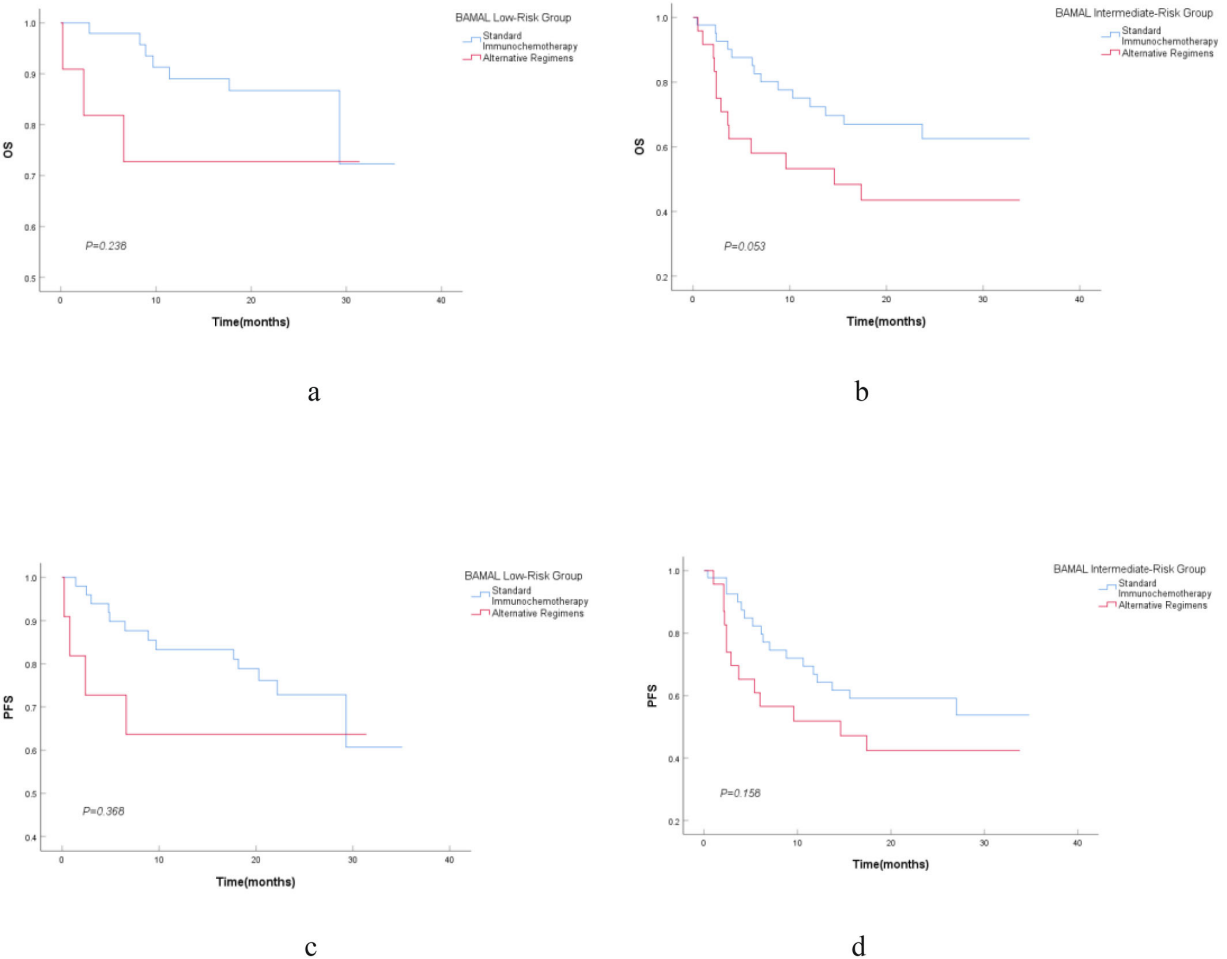
Kaplan-meier survival estimates stratified by treatment intensity within BAMAL risk groups. OS **(a)** and PFS **(c)** in the Low-Risk Group. OS **(b)** and PFS **(d)** in the Intermediate-Risk Group. OS, Overall Survival; PFS, Progression-Free Survival.

Survival outcomes were subsequently analyzed to evaluate the impact of treatment intensity within each risk stratum (**Table 5**, **Figure 3**). In the Low-Risk group, no statistically significant difference was observed between patients receiving standard versus alternative regimens in terms of Overall Survival (OS, *P = 0.238*) or Progression-Free Survival (PFS, *P = 0.368*). This lack of difference likely reflects the excellent baseline prognosis of this subgroup or potential selection bias, where alternative regimens were reserved for patients with very limited disease burden. Conversely, in the Intermediate-Risk group, treatment intensity appeared to have a clinically relevant impact. Patients receiving standard immunochemotherapy demonstrated a trend toward superior OS compared to those receiving alternative regimens (*P = 0.053*). The median OS for the alternative treatment group was notably poor at 14.6 months (95% CI: 0.000–30.775), whereas the median OS for the standard treatment group was not reached. A similar trend was observed for PFS (*P = 0.158*), suggesting that for intermediate-risk patients, unwarranted treatment de-escalation may compromise long-term survival.

**Table 5:** Subgroup analysis of overall survival and progression-free survival by treatment regimen across BAMAL risk groups.

Risk stratification and treatment	No. of patients (N)	Overall survival (OS)			Progression-free survival (PFS)		
		Events	Median (95% CI)	*P* value	Events	Median (95% CI)	*P* value
Low-risk group	62	10	NA(NA-NA)	*0.238*	17	NA(NA-NA)	*0.368*
Standard immunochemotherapy	51	7	NA(NA-NA)		13	NA(NA-NA)	
Alternative regimens	11	3	NA(NA-NA)		4	NA(NA-NA)	
Intermediate risk	68	27	NA(NA-NA)	*0.053*	30	27.000(NA-NA)	*0.158*
Standard immunochemotherapy	43	14	NA(NA-NA)		17	NA(NA-NA)	
Alternative regimens	25	13	14.600(0.000-30.775)		13	14.600(0.000-31.273)	
High-risk group	6	5	NA(NA-NA)	NA	5	NA(NA-NA)	NA
Standard immunochemotherapy	1	0	NA(NA-NA)		0	NA(NA-NA)	
Alternative regimens	5	5	NA(NA-NA)		5	NA(NA-NA)	

CI, Confidence Interval; NA, Not Reached (or Not Available). *P* values were calculated using the Log-rank test.

**Figure 4:**
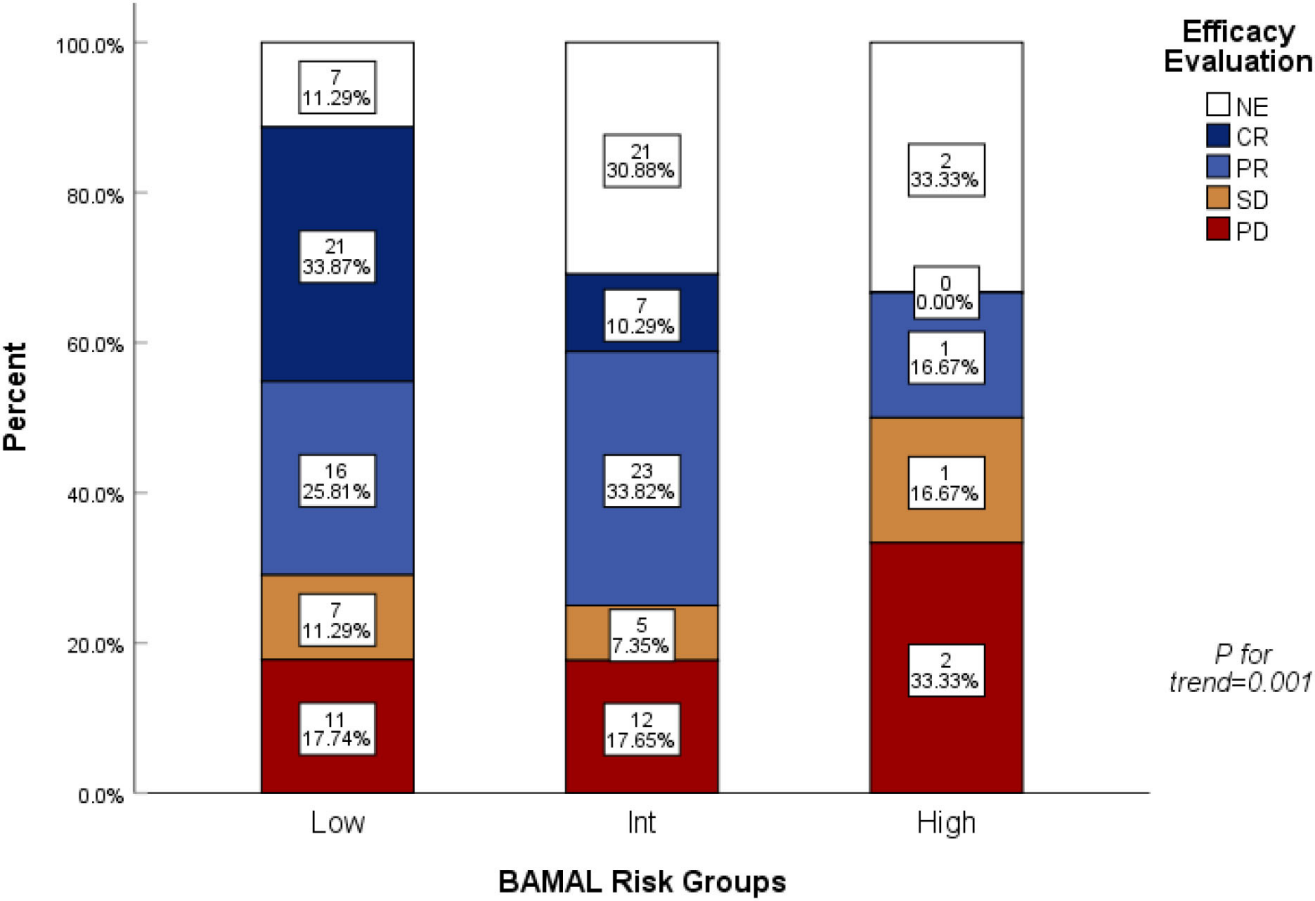
Stacked bar chart illustrating the linear association between BAMAL risk categories and therapeutic response rates. CR, Complete Response; PR, Partial Response; SD, Stable Disease; PD, Progressive Disease; NE, Not Evaluable. *P* values were calculated using the Chi-square test or Fisher’s exact test to assess the trend across risk groups.

Outcomes for the High-Risk group (n=6) were uniformly poor, characterized by high rates of treatment failure and competing mortality risks. While statistical comparison was not feasible due to the small sample size, a detailed examination of individual patient outcomes provided critical insights. Among the five patients receiving alternative regimens (R-CVP or R-monotherapy), all experienced rapid treatment failure: three died from lymphoma progression (median time to progression < 6 months), one died from severe pneumonia at 5 months, and one died from heart failure immediately following treatment. These events underscore the dual burden of aggressive disease biology and severe physiological frailty (high mFI-5 and AST) in this population. Notably, the single high-risk patient who tolerated standard R-CHOP achieved SD and remained alive at the data cutoff (OS: 34 months). Analysis of therapeutic response rates further elucidated the drivers of these survival disparities (**Figure 4**). A profound “response gradient” was observed: the Overall Response Rate (ORR, CR+PR) plummeted from 59.7% in the Low-Risk group to 16.7% in the High-Risk group (*P for trend = 0.001*). Most critically, the rate of CR—the strongest predictor of long-term cure—vanished entirely in the High-Risk cohort (0%), compared to 33.9% in Low-Risk patients. Furthermore, the proportion of patients deemed “Not Evaluable” (NE) rose sharply with increasing risk (Low: 11.3% vs. High: 33.3%), reflecting a high incidence of early treatment abandonment or rapid clinical deterioration before efficacy could be assessed.

### 3.4 Prognostic model comparison

In this patient cohort, traditional prognostic models (IPI, R-IPI, and NCCN-IPI) all demonstrated the ability to significantly differentiate between patients of different risk levels in terms of OS (all *P<0.05*). To rigorously evaluate the predictive superiority of the novel BAMAL score, we performed a multi-dimensional comparison involving discrimination, model fit, calibration, and clinical net benefit.

**Figure 5:**
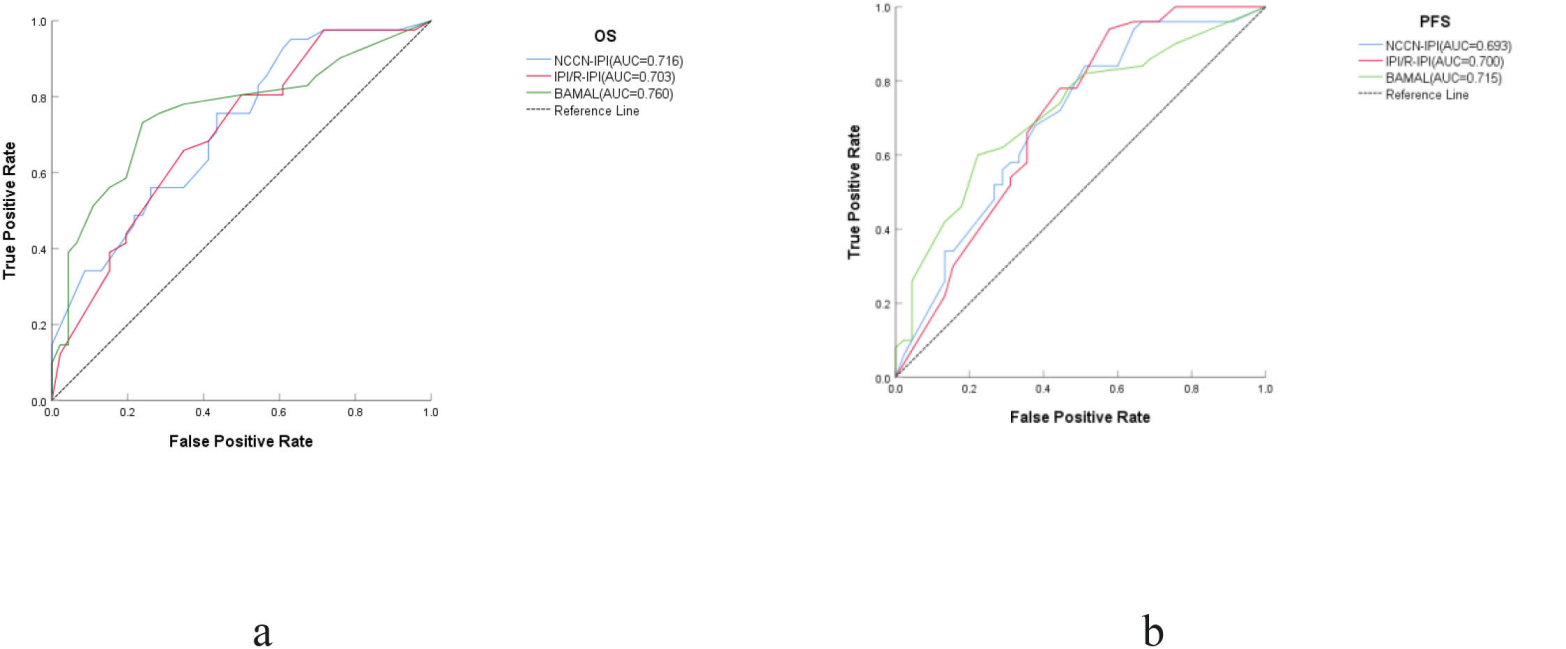
Receiver operating characteristic (ROC) curves comparing the predictive performance of the BAMAL, NCCN-IPI, and IPI/R-IPI models for Overall Survival (OS) and Progression-Free Survival (PFS). **(a)** OS; **(b)** PFS. AUC, Area Under the Curve; IPI, International Prognostic Index; R-IPI, Revised-IPI; NCCN-IPI, National Comprehensive Cancer Network-IPI.

Regarding discriminatory performance, the BAMAL score yielded the highest numerical time-dependent AUC for overall survival (0.760; 95% CI, 0.655–0.865), surpassing both the NCCN-IPI (0.716; 95% CI, 0.609–0.822) and the IPI/R-IPI (0.703; 95% CI, 0.594–0.812) (**Figure 5**). To determine if these observed differences were statistically significant, we employed the DeLong test for paired ROC curves. The analysis revealed that the differences in AUC between the BAMAL score and the traditional indices did not reach statistical significance (BAMAL vs. NCCN-IPI: *P=0.358*[-0.050-0.138]; BAMAL vs. IPI/R-IPI: *P=0.268*[-0.044-0.158]). Similarly, for PFS, the BAMAL score achieved a time-dependent AUC of 0.715(0.612-0.819), which was comparable to the NCCN-IPI (AUC = 0.693[0.586-0.800]; *P=0.612*[-0.064-0.109]) and IPI/R-IPI (AUC = 0.700[0.592-0.807]; *P=0.675*[-0.058-0.090]). These results indicate that while the BAMAL score does not statistically outperform traditional models in pure discriminatory power, it is non-inferior and possesses comparable predictive accuracy in this elderly cohort. However, discriminatory power alone does not capture the full quality of a prognostic model. Assessment via the AIC showed that the BAMAL score yielded the lowest AIC values for both OS (366.5) and PFS (460.2), significantly lower than those of the NCCN-IPI (OS: 446.9; PFS: 461.2) and IPI/R-IPI (OS: 378.0; PFS: 463.8). This lower AIC indicates that the BAMAL score achieves the optimal balance between model parsimony and explanatory power, minimizing information loss more effectively than the standard indices.

**Figure 6:**
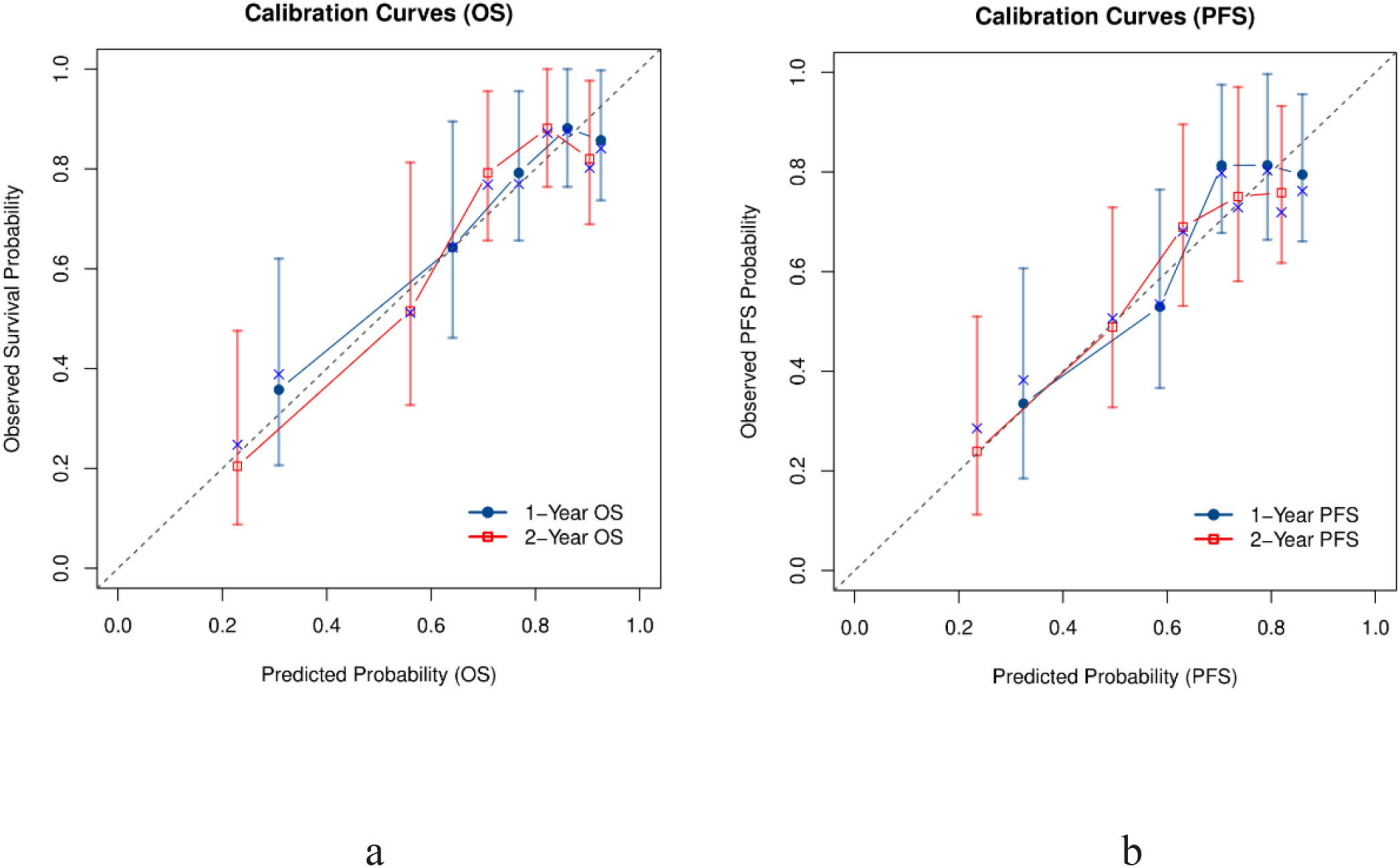
Calibration curves of the BAMAL score for survival prediction. **(a)** 1-year OS and 2-year OS; **(b)** 1-year PFS and 2-year PFS. The diagonal dashed line represents ideal prediction. OS, Overall Survival; PFS, Progression-Free Survival.

We further evaluated the accuracy of the BAMAL score through calibration analysis. The calibration curves for 1-year and 2-year OS and PFS demonstrated excellent agreement between the predicted survival probabilities and the actual observed outcomes (**Figure 6**). Visually, the curves for both endpoints closely tracked the ideal 45-degree diagonal line, indicating that the model provides reliable risk estimates across the entire risk spectrum. Specifically, for the primary endpoint of OS, the model maintained robust calibration without significant overestimation or underestimation, confirming its reliability for mortality risk stratification in this heterogeneous elderly population.

**Figure 7:**
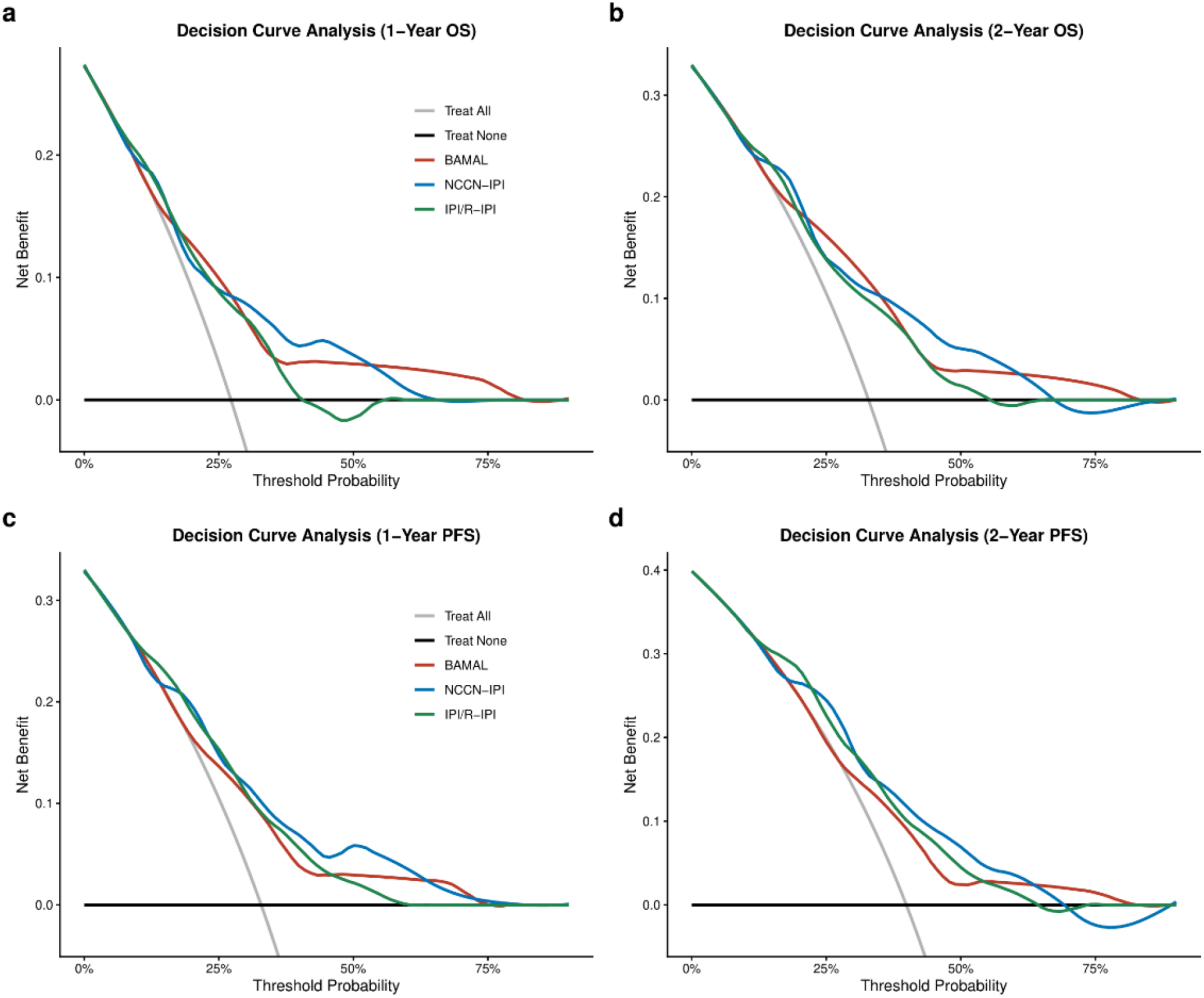
Decision Curve Analysis (DCA) of the BAMAL score versus traditional models. **(a)** 1-year OS; **(b)** 2-year OS; **(c)** 1-year PFS; **(d)** 2-year PFS. The y-axis represents the net benefit calculated by subtracting the proportion of false positives from the proportion of true positives, weighted by the relative harm of false-positive and false-negative results. OS, Overall Survival; PFS, Progression-Free Survival.

Finally, to assess the practical value of the model in clinical decision-making, we conducted DCA. The analysis revealed distinct patterns of clinical utility between endpoints, with the BAMAL score demonstrating a more robust net benefit for Overall Survival (OS) compared to Progression-Free Survival (PFS) at both 1-year and 2-year time points. For 1-year prediction endpoints, the BAMAL score provided a clinical net benefit comparable to the NCCN-IPI and IPI/R-IPI across most reasonable threshold probabilities. However, for 2-year OS—a critical landmark for elderly survival—the BAMAL score exhibited superior net benefit, particularly in the high-threshold probability range (> 60%), where traditional indices offered negligible utility. (**Figure 7**). In contrast, while the model showed positive net benefit for PFS, the magnitude of benefit was less pronounced than for OS, further validating the model’s primary strength in predicting holistic survival outcomes. The BAMAL score, by incorporating host vulnerability factors (mFI-5 and AST), maintained a positive net benefit in this high-risk zone, suggesting it may be a more valuable tool for identifying patients who would benefit from alternative or de-escalated management strategies rather than standard aggressive immunochemotherapy.

## 4 Discussion

The clinical management of elderly patients with DLBCL is complex, requiring a delicate balance between achieving a curative response and minimizing treatment-related toxicity. This study successfully developed and internally validated a novel, practical, and robust prognostic model—the BAMAL score—that not only provides superior model fit and calibration compared to the standard NCCN-IPI but also offers critical insights into treatment selection and “competing mortality” risks in real-world practice. The core strength of the BAMAL score lies in its fundamental design, which achieves an effective integration of both tumor-centric characteristics and host-related vulnerability factors.

Traditional prognostic models, like the IPI, primarily focus on tumor-related metrics such as tumor stage and LDH level, which reflect the aggressiveness and extent of the malignancy. However, the data from this study indicates that this approach is insufficient for the elderly. A remarkable 35.7% of all patient deaths were attributed to non-lymphoma-related causes, with pulmonary infections being the most common, followed by cardiovascular failure. This finding highlights a critical clinical reality: for older patients, survival is not only determined by the aggressive behavior of the lymphoma but is also heavily influenced by their overall physiological vulnerability and susceptibility to “competing mortality risks” (2, 9). The BAMAL score directly addresses this critical gap by incorporating parameters that quantify the host’s intrinsic state, thereby providing a more accurate reflection of the patient’s capacity to withstand the rigors of therapy and to survive non-oncological complications.

A critical methodological decision in this study was to prioritize OS as the primary endpoint for model development. While we identified significant predictors for PFS (e.g., double-expressor status), in geriatric oncology, OS is considered the most robust endpoint because it captures the composite impact of both tumor aggressiveness (progression) and host vulnerability (treatment toxicity and non-lymphoma mortality) (45, 46). Our cohort revealed a high rate of non-lymphoma mortality (35.7%), underscoring that for elderly patients, mortality is driven not solely by lymphoma progression but also significantly by comorbidities and treatment toxicity (e.g., infection, heart failure) (47, 48). Constructing the model based on OS ensures that the score reflects the patient’s holistic prognosis—answering the critical question of “who will survive”, rather than solely “whose tumor will respond”. Although this limits the model’s sensitivity in predicting pure biological progression (PFS), it enhances its clinical utility for decision-making in an elderly population with competing risks. A key component of this comprehensive assessment is the inclusion of the modified frailty index (mFI-5). The limitations of the conventional ECOG PS score are well-documented. It is a subjective measure that often fails to fully capture the complex, multi-dimensional nature of frailty in older patients (2, 5). A previous study on elderly cancer patients demonstrated that ECOG PS correlates poorly with comorbidities, with a low AUC of 0.55 (49). This means that a patient can have a relatively good ECOG PS score while possessing significant underlying health deficits that compromise their resilience. The fact that ECOG PS lost its independent prognostic value in the multivariate analysis in our study, while mFI-5 retained it, provides strong evidence for this point. The mFI-5 score, by objectively assessing functional status and comorbidities, provides a more reliable measure of a patient’s biological age and physiological reserve, enabling a more accurate prediction of treatment tolerance, risk of toxicity, and overall survival.

The individual components of the BAMAL score each hold significant clinical and biological meaning. Bone marrow involvement and elevated LDH are well-established adverse prognostic factors in DLBCL, reflecting high tumor burden and rapid cellular proliferation (50, 51). Their prognostic value was re-confirmed in our study. The novel aspects of the BAMAL score are its systematic quantification of host vulnerability.

Complementing the assessment of physiological frailty, the inclusion of elevated AST as an independent adverse prognostic factor is particularly noteworthy. While elevated AST can reflect liver infiltration by lymphoma cells, its prognostic value may extend beyond simple organ involvement (15). A study by Solmaz et al. found that a higher AST/ALT ratio was an independent adverse prognostic factor for OS in DLBCL patients, suggesting a systemic rather than localized effect (52). In our study, AST retained its independent prognostic value even after accounting for other factors, suggesting it may serve as a simple, objective biomarker for a more aggressive tumor metabolic phenotype or a generalized systemic stress response (15, 52). This finding suggests that AST could act as a “silent signal” of a deeper physiological distress or a more aggressive tumor-host interaction, providing a window into a patient’s unique biological state that is not captured by other standard measures.

Beyond prognostication, the BAMAL score demonstrated significant potential to guide therapeutic decision-making. We observed a strong inverse correlation between BAMAL risk stratification and the utilization of standard immunochemotherapy (*P for trend < 0.001*), confirming that the score parallels clinical judgment. However, our subgroup survival analysis revealed nuanced implications for treatment optimization. In the Intermediate-Risk Group, patients receiving standard immunochemotherapy (including R-CHOP and R-mini-CHOP) exhibited a strong trend toward superior overall survival compared to those receiving Alternative Regimens (e.g., R-CVP, CHOP) (*P = 0.053*, Median OS: Not Reached vs. 14.6 months). This finding is clinically pivotal. It corroborates recent real-world evidence suggesting that omitting anthracyclines or rituximab in “unfit” patients frequently leads to inferior outcomes (47, 51). Our data suggest that for BAMAL intermediate-risk patients, the therapeutic goal should be “optimization” (e.g., using R-mini-CHOP) rather than “avoidance” of standard therapy. De-escalation to non-anthracycline regimens should be reserved strictly for those with absolute contraindications.

Conversely, in the High-Risk Group, prognosis was uniformly dismal regardless of treatment intensity, with a high mortality rate driven by both rapid disease progression and severe frailty. This dismal trajectory is mechanically explained by our response analysis: the complete absence of CR in this subgroup suggests that standard cytotoxic agents cannot clear the disease burden in the face of such physiological vulnerability. The high rate of “Not Evaluable” outcomes further corroborates that these patients often succumb to toxicity or functional collapse before the anti-tumor effect can manifest. This indicates that for this distinct subgroup of highly vulnerable patients, current cytotoxic strategies—whether intensive or attenuated—are largely futile. This observation highlights an urgent unmet medical need and supports the exploration of chemotherapy-free approaches, such as bispecific antibodies (e.g., epcoritamab, glofitamab) or BTK inhibitors, which may offer disease control without the prohibitive toxicity of traditional chemotherapy (53–56).

Complementing its robust prognostic discrimination, the BAMAL score demonstrated satisfactory calibration and clinical utility. The high concordance between predicted and observed rates for both OS and PFS confirms that the model provides realistic risk estimates, avoiding the common pitfalls of overfitting often seen in small cohorts. This reliability in risk estimation lays the foundation for its clinical utility. Decision Curve Analysis (DCA) further substantiated the clinical utility of the BAMAL score, highlighting a notable performance advantage for OS over PFS. This divergence aligns with our model’s construction, which captures competing mortality risks that terminate OS but do not necessarily manifest as lymphoma progression (PFS). The score’s value was most evident for 2-year OS in the high-risk threshold probability range (> 60%). In this range, where clinicians face the most difficult dilemma—weighing the burden of toxic treatment against the likelihood of futility—the BAMAL score provides a more reliable tool to identify patients who are truly “too frail to treat” with standard protocols, thereby avoiding futile toxicity while ensuring that fit patients are not denied potentially curative therapy.

Methodologically, the construction of the BAMAL score prioritized clinical utility, necessitating the dichotomization of continuous variables based on literature-validated cut-offs. While this transformation inevitably resulted in statistical information loss and attenuated P-values for certain components (e.g., Age and LDH) due to sample size constraints, the robustness of these factors was rigorously confirmed by the preliminary backward stepwise regression incorporating continuous variables. Furthermore, the statistical overshadowing of chronological age by mFI-5 in the categorical model offers a critical biological insight: physiological frailty is a superior predictor of survival compared to chronological age in the elderly. Ultimately, this modeling strategy successfully balances statistical rigor with the practical need for a simplified, user-friendly tool in clinical settings.

This study has several limitations that warrant consideration, and our findings should be interpreted as exploratory given the retrospective, single-center design and the relatively limited sample size (n=136). These factors may introduce selection bias and limit the generalizability of the findings. The relatively limited sample size could affect the power of some subgroup analyses. Additionally, the dichotomization of continuous predictors for score construction inevitably resulted in statistical information loss, contributing to the attenuated significance observed for certain model components. The median follow-up time of 25.1 months is sufficient for assessing 2-year survival but may be inadequate for evaluating long-term outcomes. Furthermore, our analysis did not employ competing risk models, which could have provided a more nuanced assessment of lymphoma-related mortality versus non-lymphoma-related mortality. Therefore, the BAMAL score requires rigorous external validation in larger, multi-center prospective cohorts to confirm its reproducibility and clinical utility. Future studies should also explore the integration of molecular markers (e.g., circulating tumor DNA) into the model to further enhance its precision for the era of precision medicine.

## 5 Conclusion

In this retrospective cohort study, we successfully developed and internally validated a novel five-factor prognostic model for newly diagnosed elderly DLBCL patients, the BAMAL score. This model uniquely integrates established tumor biological characteristics (bone marrow involvement and LDH level) with crucial host-related parameters (mFI-5 score, age ≥75 years, and AST level). The BAMAL score outperformed standard indices and provided actionable guidance for personalized treatment: supporting the use of standard immunochemotherapy in intermediate-risk patients while identifying high-risk patients who are candidates for novel, non-cytotoxic clinical trials.

## Data Availability

The original contributions presented in the study are included in the article/Supplementary Material. Further inquiries can be directed to the corresponding authors.
